# Skeletal muscle redox signaling in rheumatoid arthritis

**DOI:** 10.1042/CS20190728

**Published:** 2020-11-04

**Authors:** Maarten M. Steinz, Estela Santos-Alves, Johanna T. Lanner

**Affiliations:** Department of Physiology and Pharmacology, Molecular Muscle Physiology and Pathophysiology laboratory, Karolinska Institute, Stockholm, Sweden

**Keywords:** muscle weakness, oxidative stress, reactive oxygen species, rheumatoid arthritis, skeletal muscle

## Abstract

Rheumatoid arthritis (RA) is a chronic inflammatory disease characterized by synovitis and the presence of serum autoantibodies. In addition, skeletal muscle weakness is a common comorbidity that contributes to inability to work and reduced quality of life. Loss in muscle mass cannot alone account for the muscle weakness induced by RA, but instead intramuscular dysfunction appears as a critical factor underlying the decreased force generating capacity for patients afflicted by arthritis. Oxidative stress and associated oxidative post-translational modifications have been shown to contribute to RA-induced muscle weakness in animal models of arthritis and patients with RA. However, it is still unclear how and which sources of reactive oxygen and nitrogen species (ROS/RNS) that are involved in the oxidative stress that drives the progression toward decreased muscle function in RA. Nevertheless, mitochondria, NADPH oxidases (NOX), nitric oxide synthases (NOS) and phospholipases (PLA) have all been associated with increased ROS/RNS production in RA-induced muscle weakness. In this review, we aim to cover potential ROS sources and underlying mechanisms of oxidative stress and loss of force production in RA. We also addressed the use of antioxidants and exercise as potential tools to counteract oxidative stress and skeletal muscle weakness.

## Rheumatoid arthritis does not only affect the joints

### Rheumatoid arthritis

Rheumatoid arthritis (RA) is one of the most common chronic inflammatory diseases that more frequently afflicts women than men, with a disease debut around the age of 40 to 50 years [1–3]. The disease is characterized by a chronic inflammation of the joints with systemically elevated levels of circulating cytokines (e.g. tumor necrosis factor-α, TNF-α; interleukin-1 and -6, IL-1 and IL-6; monocyte chemoattractant protein 1, MCP1; oncostatin M, OSM), autoantibodies to immunoglobulin G (i.e. rheumatoid factor, RF) and autoantibodies against citrullinated proteins (i.e. anti-citrullinated protein antibodies, ACPAs) [2,4]. Muscle weakness is a recurrent complication for patients with rheumatoid arthritis (RA) that may reduce their quality of life [5–7]. Patients with RA suffer from muscle weakness in addition to the primary inflammation of the joints [5,7–12]. Moreover, tight disease control with anti-rheumatic drugs that target the inflammation has been shown insufficient to counteract muscle weakness in patients with RA [9]. The molecular details behind RA-induced muscle weakness are still not fully known, but in this review we aim to cover the current status in the field of how RA contributes to the onset of muscle weakness and how this may be counteracted.

### Muscle strength and muscle weakness

#### Muscle force

**Figure 1:**
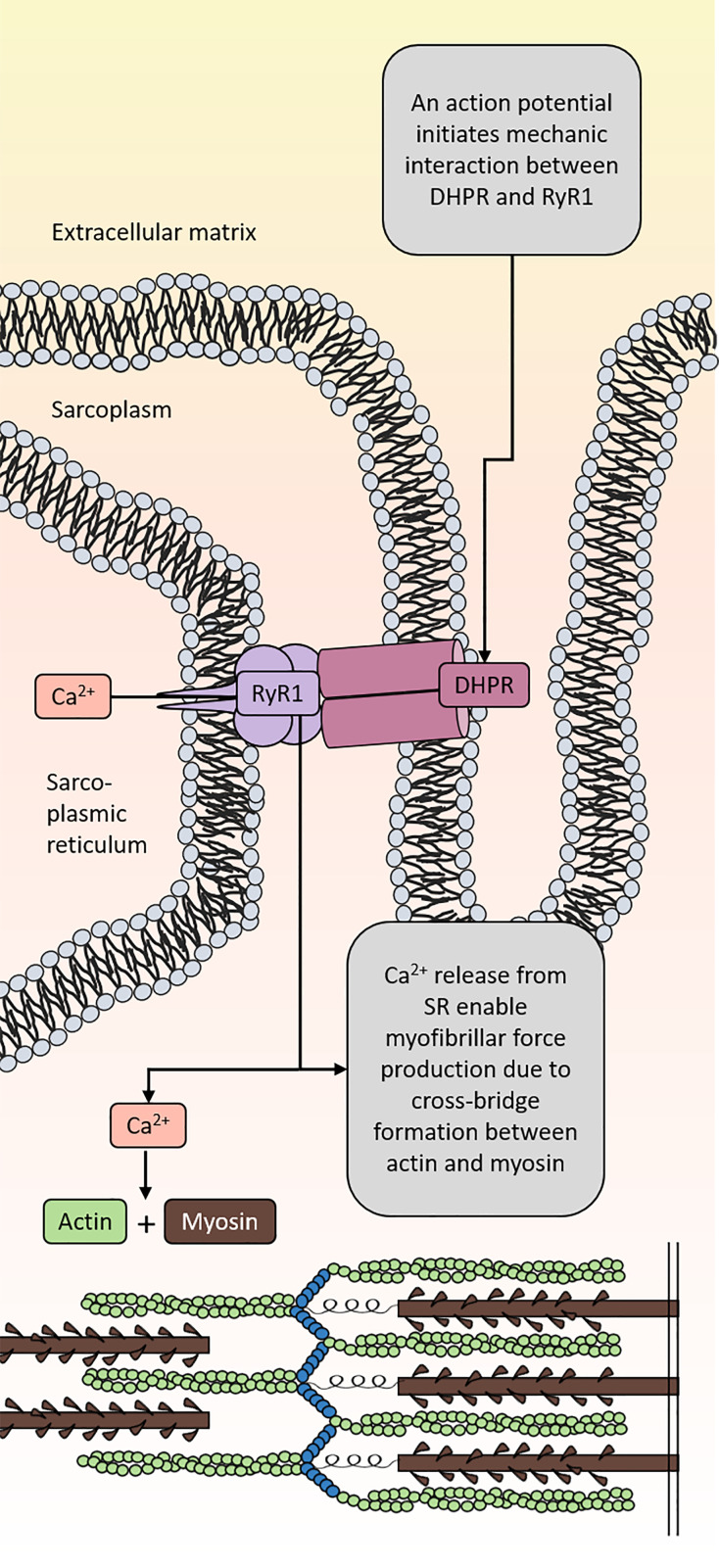
Excitation-contraction coupling (ECC) ECC starts with an action potential that reaches the sarcolemma and continues down the t-tubular system of the muscle where it depolarizes and thus activates the dihypropyridine receptors (DHPR/Cav1.1). Activated DHPR mechanically interacts with RyR1 in the SR membrane. The interaction results in RyR1 activation and Ca^2+^ release from SR. Elevated Ca^2+^ levels in the myoplasm enable actin and myosin binding that leads to force production. Upon relaxation, Ca^2+^ is pumped back into the SR through the SR Ca^2+^ ATPase (SERCA, not shown in this figure).

The human body consists of ∼35–40% of skeletal muscle relative to body weight [13]. Skeletal muscle is essential for our ability to move and one of its major functions is to produce strength, i.e. muscle force [14,15]. The muscle consists of bundles of myofibers comprising thousands of myofibrils as the functional unit for muscle force production [15]. The myofibrils are a repeated organization of thick and thin filaments that contain the contractile proteins myosin and actin, respectively, which form cross-bridges for muscle force production [16]. Cross-bridge formation between actin and myosin requires calcium (Ca^2+^) release from the sarcoplasmic reticulum (SR) located inside the muscle cell [17,18]. The release of Ca^2+^ from the SR into the muscle cytoplasm is regulated by the ryanodine receptor 1 (RyR1), which is a large channel located in the SR membrane. RyR1 activity is regulated by many proteins (e.g. DHPR/Cav1.1, FKBP12, calmodulin), ions (Ca^2+^, Mg^2+^) and post-translational modifications (e.g. nitrosylation, carbonylation, phosphorylation) [17,19,20]. Excitation-contraction coupling (ECC) is the sequence of events that starts with an action potential in the α-motor neuron that leads to activation of RyR1, Ca^2+^ release and actin–myosin interaction for generation of force (Figure 1) [17]. In addition, the actin-bound troponin–tropomyosin complex and the sarco/endoplasmic reticulum Ca^2+^-ATPase (SERCA) are also important muscle proteins for muscle contraction [17] (but not further discussed in this review).

#### Muscle weakness in RA

In 2015, van Vilsteren et. al. showed that physical limitations afflicting RA patients were significantly associated with a reduced work productivity [7]. Furthermore, in a study including >5000 RA patients it was shown that more than 80% does not regularly exercise despite the fact that regular exercise is associated with better physical health [6]. There might be several reasons behind the observed physical limitation, but muscle weakness is considered as a dominating factor [13]. Muscle weakness refers to a decrease in muscle strength and in RA patients it has often been attributed to a decrease in muscle mass (i.e. atrophy and cachexia) [21,22]. However, Helliwell and Jackson reported already in 1994 that only ∼50% of the reduction in fore-arm grip strength of RA patients could be explained by a decreased muscle size [8]. More recent studies have also concluded that arthritis-induced muscle weakness cannot solely be explained by a decrease in muscle mass, but instead that intramuscular changes may be the underlying factors of decreased force generating capacity for patients afflicted by arthritis [5,9–11,23,24]. For instance, electron microscopy analyses of muscle biopsy samples have shown that muscles from RA patients present more intramuscular alterations than muscles from healthy control subjects with, for example, wider separation between myofibrils, dilated t-tubular system, pleomorphic mitochondria, myofibrillar flaking and lipofuscin deposition in the subsarcolemmal region [10]. Moreover, post-translational modifications on important contractile proteins accompanying the arthritis-induced muscle weakness have been observed in rodent models of arthritis [23,24]. Recently, we also showed that oxidative stress-induced post-translational modifications on the contractile protein actin results in decreased ability of actin and myosin to form force-generating cross-bridges and thereby directly contribute to muscle weakness in a mouse model of arthritis and in patients with RA [11]. Thus, RA appears to induce both muscle atrophy and intrinsic muscle dysfunction which leads to reduced force production and muscle weakness.

## Oxidative stress and muscle weakness in RA

Oxidative stress is used to describe the maladaptive effects of an imbalance between production and scavenging of reactive oxygen and nitrogen species (ROS/RNS) [25]. Examples of ROS are superoxide (O_2_^•−^), peroxynitrite (ONOO^−^) and hydroxyl radicals (OH^•^) or non-free radicals such as hydrogen peroxide (H_2_O_2_) [26]. The half-life of ROS/RNS is very short (i.e. ONOO^−^ ∼5-20 ms; O_2_^−•^, ∼1 μs). Therefore, the occurrence of oxidative stress has often been assessed by markers of oxidative stress, for example, measuring oxidative post-translational modifications (oxPTMs) [11,12,27]. There are several types of oxPTMs, for example, nitrosylation (SNO), carbonylation (DNP), nitration (3-NT) and malondialdehyde (MDA) adducts [28]. Several studies have shown that oxidative stress is part of the pathology of RA and is associated with the onset of RA-induced muscle weakness [23,24,27,29,30]. For example, increased levels of antibodies against MDA adducts have been found in blood of patients with RA [27]. Moreover, studies have shown increased levels of 3-NT and MDA modifications on muscle proteins in association with muscle weakness in rodents with arthritis and patients with RA [11,12,23,24]. Actin with its essential function for force production has been of major interest for linking oxPTMs to muscle weakness in RA [11,23,24,31]. For instance, in muscle homogenates from rats with adjuvant-induced arthritis, Yamada and colleagues observed the formation of actin aggregates of ∼150 kDa, which were enriched with 3-NT and MDA adducts [23]. Interestingly, antioxidant treatment with a SOD/catalase mimetic resulted in a reduced amount of 3-NT and MDA adducts on actin, less actin aggregate formation and counteracted arthritis-induced muscle weakness [23]. More recently, we also have identified oxPTMs in three specific hotspots of actin in adjuvant-induced arthritis mice and patients with RA [11]. These modifications reduced the ability of actin to polymerize and decreased its ability to form force-generating cross-bridges [11]. Thus, 3-NT and MDA on skeletal muscle actin contribute to arthritis-induced muscle weakness.

RyR1 and its role in muscle weakness has gained a lot of interested over the years [19,20]. For example, SNO modification of cysteines and carbonylation of RyR1 have been shown to make the channel less stable and thus lead to increased open probability of the channel, which have been observed in muscle dysfunction associated with, for example, malignant hyperthermia, bone metastases, Duchenne muscular dystrophy, heart failure and normal aging [32–37]. Moreover, Yamada et al. (2015) showed that 3-NT modifications on the RyR1 macromolecular complex were associated with decreased muscle force in collagen-induced arthritis mice [24]. However, the occurrence and relevance of specific oxPTMs on RyR1 in association with RA-induced muscle weakness is unknown.

## Sources of oxidative stress in skeletal muscle

Although oxidative stress is linked to muscle weakness in RA, it is still unclear how arthritis affects the sources and scavengers of ROS/RNS in skeletal muscle. Nevertheless, mitochondria, NADPH oxidases (NOX), nitric oxide synthases (NOS) and phospholipases (PLA) have all been associated with increased ROS production in RA-induced muscle weakness.

### Mitochondria

**Figure 2:**
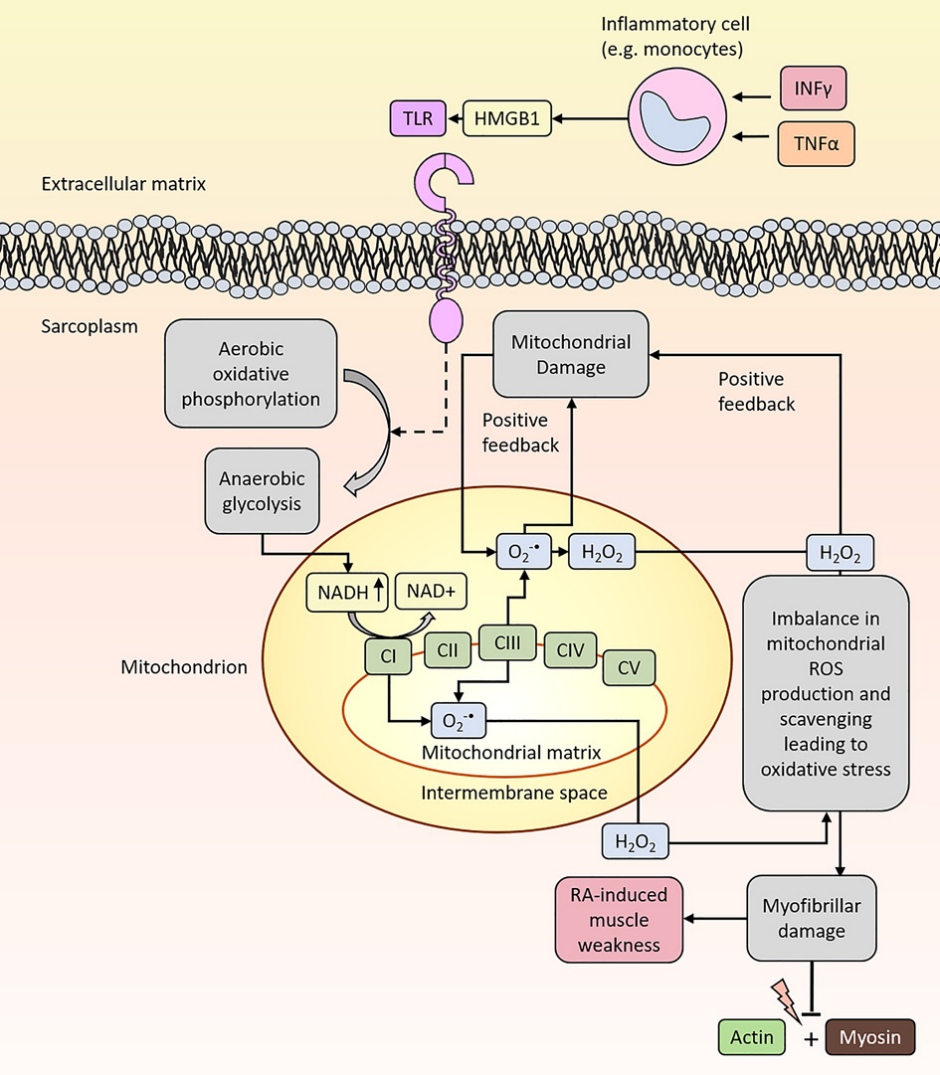
Mitochondria produce O_2_^•-^ and H_2_O_2_ which can contribute to the onset of oxidative stress in the muscle RA patients have increased circulating levels of inflammatory cytokines (e.g. IFNy, TNFα and HMGB1) which can induce ROS production. Binding of HMGB1 to toll-like receptors (TLR) located on the surface membrane can lead to mitochondrial ROS production. O_2_^•-^ is converted to H_2_O_2_ by superoxide dismutase (SOD, not shown here). H_2_O_2_ can pass through membranes of the mitochondrion and thus may elicit extra-mitochondrial oxidative stress on essential proteins for force production.

Mitochondria are essential for the production of energy (ATP) in the muscle cell [38,39]. They have a double layered membrane that comprises the intramitochondrial space and mitochondrial matrix [39]. The electron transport chain with its mitochondrial complexes are located in the inner mitochondrial membrane and can form O_2_^•−^ (primarily complex I, NADH dehydrogenase) (Figure 2) [40]. Muscle are rich in mitochondria and hence mitochondria have been considered as one potentially influential source for oxidative stress in RA-induced muscle weakness. Moreover, since mitochondria are closely located to the myofibrils, at the Z-line of the sarcomeres [41,42], they could potentially inflict oxPTMs on myofibrillar proteins. Complex I forms O_2_^•−^ through transfer of electrons by its prosthetic-group flavine mononucleotide (FMN) to O_2_ [40]. FMN accepts electrons from NADH and transfers them to coenzyme Q10 (CoQ_10_) [40]. Mitochondrial O_2_^•−^ production is associated with increases in NADH/NAD^+^ and FAD/FADH_2_ ratios as the result of changes in energy substrates, oxygen levels, ATP demands, and pro-inflammatory and oxidant environments [40,43]. Excessive ROS/O_2_^•−^ production by the mitochondria can initiate a vicious cycle, where oxidative stress interferes with the mitochondrial function and exacerbates ROS production [40]. Mitochondrial dysfunction and altered mitochondrial gene expression have been associated with arthritis, including markedly changed size and shape of mitochondria in skeletal muscles biopsies from patients with RA [10,44,45].

#### Altered mitochondrial function and ROS production in RA

Toll-like receptors (TLRs) are implicated in the onset and development of RA. McGarry et al. showed that TLR2-activated RA-synovial fibroblast cell exhibit reduced mitochondrial oxidative capacity and ATP production, as well as increased glycolytic/oxidative phosphorylation ratio [44]. Furthermore, the altered mitochondrial function was accompanied by an increase of ROS production, lipid peroxidation and mitochondrial DNA mutations. TLR-2 activation also elicited a pro-inflammatory response (e.g. secretion of IL-6 and IL-8) dependent on an energy switch from aerobic oxidative phosphorylation towards anaerobic glycolysis [44].

There are different types of ligands that can bind to TLRs, such as the high-mobility group box 1 protein (HMGB1), which is released by pro-inflammatory cells like monocytes upon stimulation by cytokines (e.g. IFN-γ and TNF-α) [46]. Concordantly, HMGB1 has been found in high concentrations in the synovium from RA patients [46]. Moreover, IFN-γ-induced accumulation of intramuscular HMGB1 have been shown to result in altered Ca^2+^ handling, potentially leading to muscle dysfunction [47]. Thus, it is plausible that the HMGB1/TLR-2 axis mediates, at least partly, the exacerbated ROS production associated with RA.

Furthermore, in a review by van Horssen, another mechanism was postulated linking increased mitochondrial ROS production and chronic inflammation. They postulated that the first step of the TCA cycle, regulated by pyruvate dehydrogenase (PDH) is a critical step in the metabolic shift mediated by pro-inflammatory cytokines [48]. Indeed, Zell and colleagues showed that 24-h exposure of cardiomyocytes to pro-inflammatory cytokines (TNF and IL-1β) reduced PDH activity in a concentration dependent manner, which was associated with a decrease in mitochondrial complex I and II activity [49]. Interestingly, high concentrations of IL-6 and pyruvate have been found in skeletal muscle of RA patients, supporting a possible cytokine-induced metabolic switch and enhanced glycolysis [50].

### NADPH oxidases

**Figure 3:**
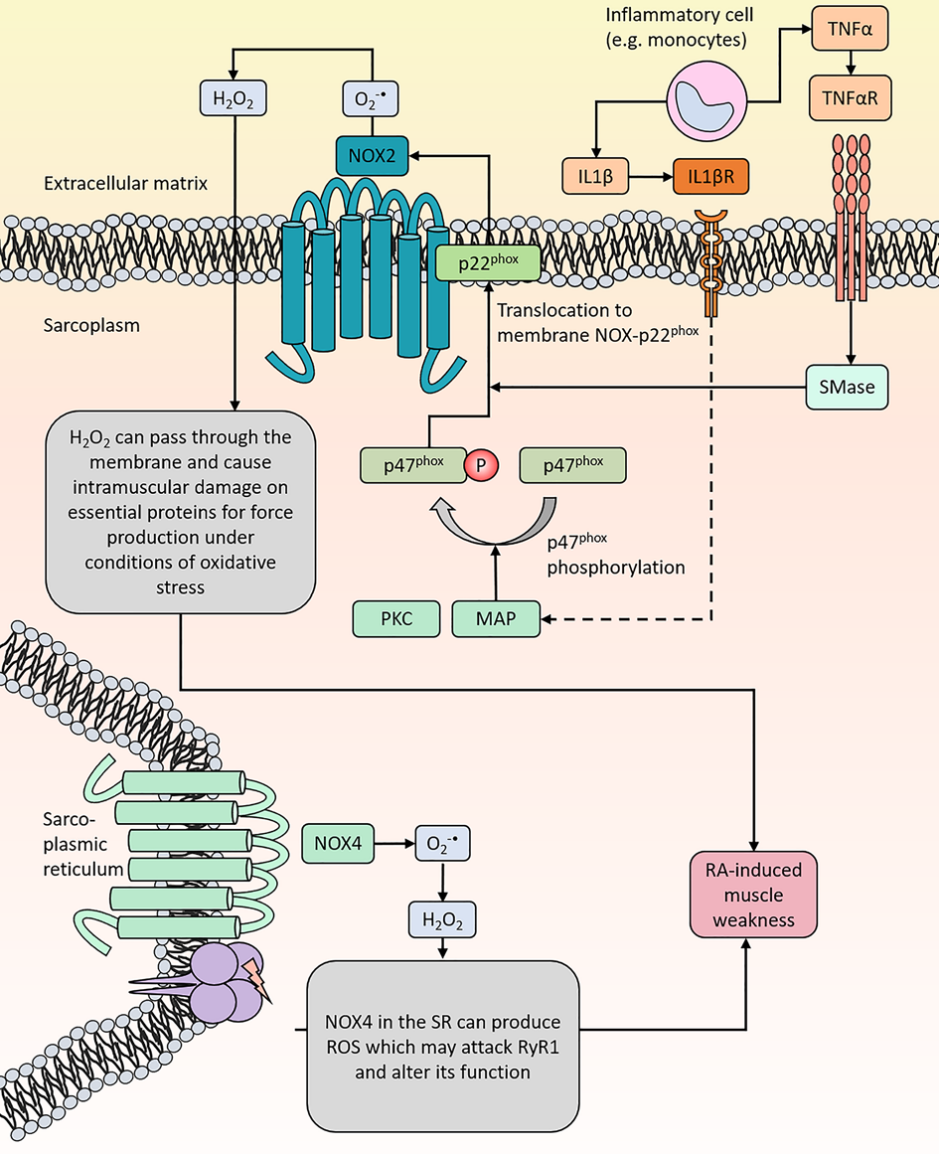
NOX redox signaling and tentative pathways for NOX-induced oxidative stress and muscle weakness RA patients have increased circulating levels of inflammatory cells (e.g. monocytes) that can release pro-inflammatory cytokines such as IL-1β and TNF-α that can activate NOX. Phosphorylation of cytosolic p47^phox^ leads to translocation together with its associated complexes (e.g. p67^phox^ and p40^phox^, here not shown) to the p22^phox^ complex, which induces O_2_^•-^ production which is rapidly converted to H_2_O_2_. NOX4 is also located in the SR membrane in close proximity to RyR1.

NADPH oxidases (NOX) are multi-subunit enzymes that can transfer electrons from NADPH to O_2_ and thereby able to form O_2_^•−^ (Figure 3), which is critical for antimicrobial host defense and regulating the innate immunity [51–53]. However, an excess NOX-induced ROS production has also been linked to cellular dysfunction, including the progression of diabetic kidney disease [54] and poor sperm function and infertility [55]. There are seven different NOX isoforms of which NOX2 and NOX4 are found in skeletal muscle [51,56,57]. Gp91^phox^ and p22^phox^ are the catalytic subunits of NOX which are membrane bound [58]. Activation of NOX requires assembly of the membrane bound subunits with the key cytosolic regulator: p47^phox^. Phosphorylation of p47^phox^ results in translocation of the cytosolic subunits that are linked to p47^phox^ (i.e. p40^phox^ and p67^phox^) toward the membrane where interaction with p22^phox^ occurs [56,58]. This will induce NOX activity causing the gp91^phox^ unit to transfer electrons from NADPH via flavin adenine dinucleotide (FAD) and heme to O_2_ resulting in O_2_^•−^ [58]. NOX2 and 4 are found in the sarcolemma [59–61] and in the invaginations of the sarcolemma into skeletal muscle (t-tubular system) [60,62]. NOX4 has also been found in the SR membrane, closely associated with RyR1 [63]. For instance, NOX4 expression and NADPH activity have been detected in RyR1 preparations from sarcoplasmic reticulum extracts [63,64]. NOX4 has also been found in the inner mitochondrial membrane [57,65,66]. siRNA-mediated knockdown of NOX4 has been shown to significantly reduces NADPH oxidase activity in purified mitochondrial fractions and blocks glucose-induced mitochondrial superoxide generation in glomerular mesangial cells from rat [65]. Whereas, up-regulation of NOX4 by hypertrophic stimuli and aging induces oxidative stress, apoptosis and cardiac dysfunction, in part because of increased mitochondrial O_2_^•−^ production and consequent oxidation of mitochondrial proteins [65].

Although the role of NOX-derived O_2_^•−^ production in RA is not well understood, it appears to play an important part. For instance, mice deficient in ph47^phox^ exhibit an aggravated pathology of arthritis [67], and increased p47^phox^ phosphorylation and O_2_^•−^ production has been found in neutrophils of synovial fluid and plasma from RA patients [68,69]. However, if or whether NOX is involved in the onset and progression of RA-associated muscle weakness is unknown. Thus far, increased expression of NOX2 has been found in skeletal muscles of rodents affected by arthritis [23], but whether that leads to increased NOX-derived O_2_^•−^ production in skeletal muscles affected by RA is not known.

As mentioned before, activation of NOX requires phosphorylation of p47^phox^ which is, among others, regulated by protein kinase C (PKC), protein kinase A and mitogen-activated protein (MAP) kinase [68,70]. Regarding the latter, Luo et al. have shown that IL-1β induces MAP kinase and nuclear factor kappa-light-chain-enhancer of activated B cells (NF-κB) mediated IL-6 expression in skeletal muscle cells [71]. NF-κB is a transcription factor involved in the expression of several genes including IL-6 and IL-8 [72]. Moreover, Henriquez-Olguin et al. have reported that IL-6 expression in skeletal muscles through NF-κB is dependent on ROS production by NOX2 [73]. In patients with RA, the levels of IL-1β and IL-6 have been observed to be increased [50,74]. However, the causative relationship between IL-1β, NF-κB, IL-6, NOX2 and ROS production in muscle afflicted by inflammation and arthritis remains to be further elucidated.

Another tentative mechanism that links increased NOX-induced O_2_^•−^ production and RA is TNF-α. This cytokine is systemically upregulated in RA [50,75] and has been associated with increased O_2_^•−^ production by NOX in human rheumatoid synovial cells and in neutrophils and monocytes from patients with RA [69,76]. In addition, TNF-α plays a role in the activation of sphingomyelinase (SMase) [77], which in turn is associated with muscle weakness via SMase-induced NOX2 activation [78,79].

### Phospholipases

Phospholipases (PLA2) are a group of enzymes that hydrolyzes membrane phospholipids at the sn-2 position to arachidonic acid (AA), which is a precursor for pro-inflammatory eicosanoids such as prostaglandins [50]. PLA2 enzymes have been found to be enhanced in the synovium of animal models with arthritis and patients with RA, and studies have highlighted their implication in the RA pro-inflammatory response [80,81]. Moreover, Duchez et al. observed lower levels of eicosanoids and reduced swelling in joints of cytosolic (c) and secreted (s) PLA2 knockout mice with k/Bxn serum-transfer arthritis [82]. Similar results were obtained by Coulthard et al. in an antigen-induced arthritis mice model, where sPLA2 inhibitors were used [83]. The activity of cPLA2 appears dependent on Ca^2+^-dependent translocation of the enzyme toward the plasma membrane and phosphorylation of serine residue 505 (Ser505) [84,85]. In line with NOX activation, phosphorylation of cPLA2^Ser505^ is thought to be phosphorylated by MAP kinases [84]. Thus, increased MAP activity may therefore not only induce NOX2-induced ROS production but may also influence PLA2-induced ROS production [86].

**Figure 4:**
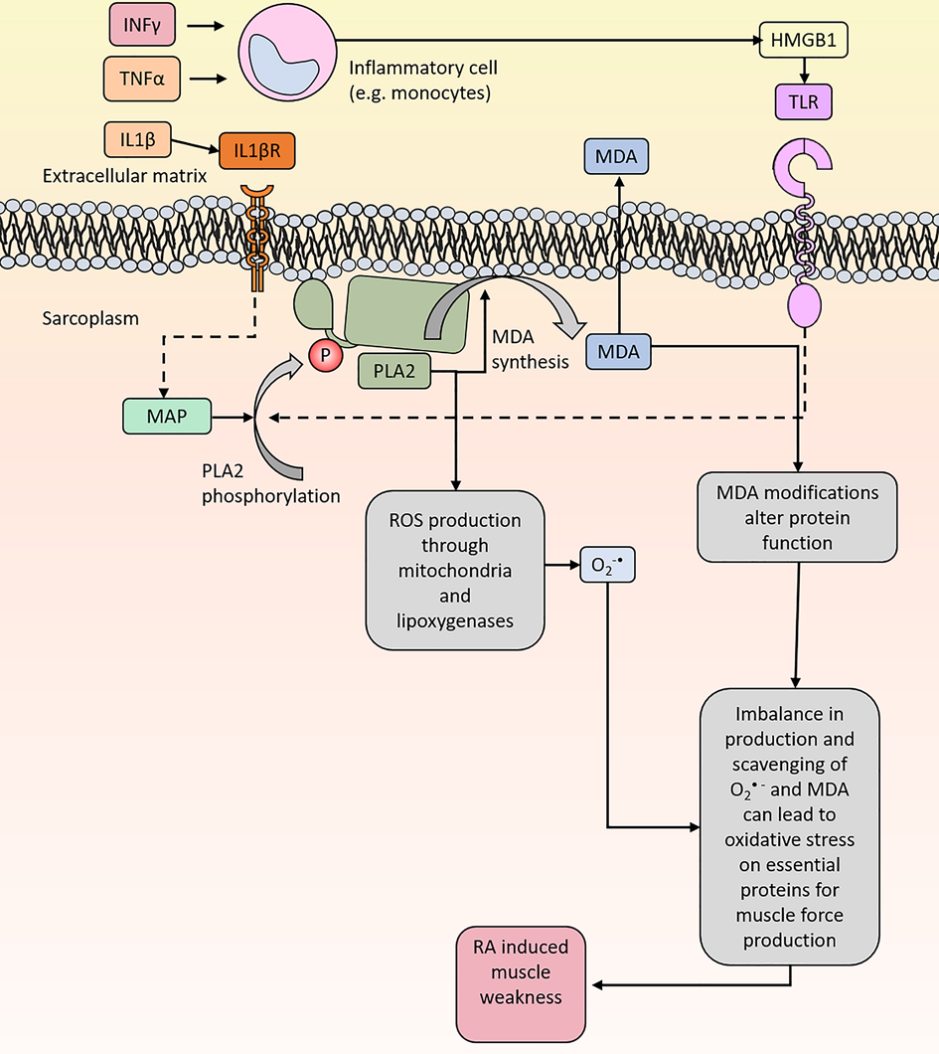
PLA2 redox signaling and the possible pathways that may lead to oxidative stress PLA2 activity can lead to O_2_^•-^ and MDA proudction which may contribute to the onset of oxidative stress and thereby contribute to muscle weakness for patients with RA. Activation of TLR receptors and IL-1β on the surface membrane can stimulate PLA2 activity. PLA2 activation is thought to be regulated by phosphorylation induced of MAP kinase.

Furthermore, oxidation by free radicals or nonradical species of AA or other unsaturated fatty acids can result in formation of MDA, among many other different aldehydes [28]. Once formed, MDA can react with proteins or DNA to form adducts resulting in biomolecular damage. Thus, enhanced PLA2 activity and AA accumulation in the presence of exacerbated O_2_^•−^ production via NOX, mitochondria or some other source can lead to elevated MDA levels and hence altered protein function (Figure 4). Indeed, increased levels of MDA and MDA-modified proteins have been observed in RA and in systemic lupus erythematous (SLE) and thus reflecting oxidative stress in these subjects [87,88]. Moreover, autoantibodies against MDA have been detected in synovial fluid from patients with RA [27]. In addition, we recently identified a set of MDA modifications on actin in muscle biopsies from patients with RA, which contributed to the exhibited muscle weakness in these patients [11].

### Nitric oxide synthases (NOS)

**Figure 5:**
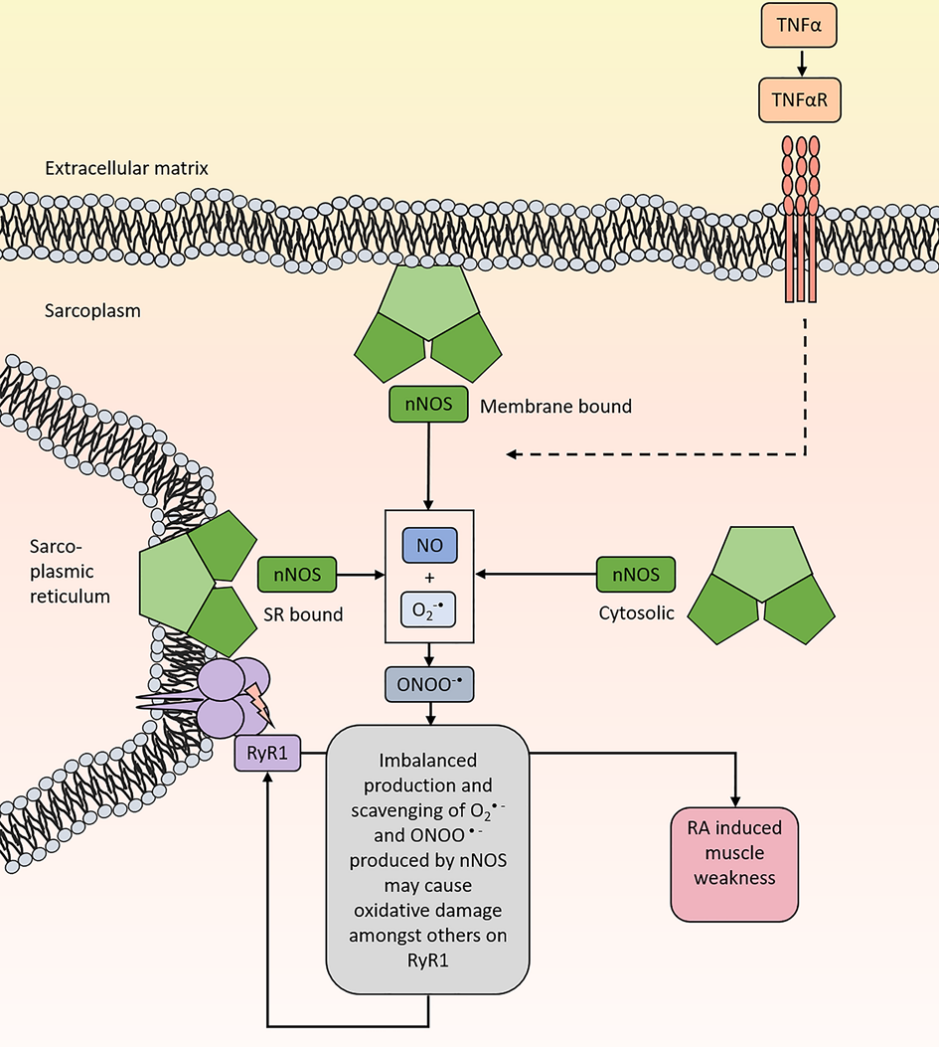
NOS redox signaling and the possible pathways that may lead to oxidative stress by induced NOS Increased circulating levels of pro-inflammatory cytokines such TNF-α have been shown to induce nNOS activity which has been linked to muscle weakness in patients with RA. The enzyme nNOS is located among other in at the sarcolemma, the membrane of the sarcoplasmic reticulum and in the cytosol of the muscles. It can produce ROS such as O_2_^−•^ and ONOO^−•^ which under oxidative stress conditions may contribute to oxidative damage on amongst others RyR1. Increased oxidative stress on the RyR1 complex mediated by nNOS has been associated with arthritis-induced muscle weakness in rodents with arthritis.

Nitric oxide synthases (NOS) are a group of enzymes that produce nitric oxide (NO) from L-arginine, NADPH and O_2_ [89–91]. There are three different isoforms of NOS expressed in skeletal muscle: neuronal NOS (nNOS, NOS1), inducible NOS (iNOS, NOS2) and endothelial NOS (eNOS, NOS3) [91]. NOS enzymes are also thought to be a source of O_2_^•-^ production that occurs when NOS is uncoupled from its substrate L-arginine and its cofactor BH4 (Figure 5) [92,93]. Thus, NOS could potentially be a source of three types of ROS/RNS, i.e. NO, O_2_^•−^, and ONOO^•−^. Among the different isoforms of NOS, increased levels and expression of nNOS have been observed in skeletal muscle from mouse models of arthritis and patients with RA [23,24]. Moreover, increased nNOS has been linked to higher levels of 3-NT modifications on the RyR1 complex skeletal muscle afflicted by arthritis-induced muscle weakness [24], and thus implying that nNOS is directly involved in adding oxPTM on RyR1. Indeed, nNOS has been shown to be expressed in the SR membrane [92] and co-localizes with RyR1 [94].

ROS/RNS production by nNOS in skeletal muscle is also thought to be regulated by TNF-α. Stasko et al. have shown a decline in skeletal muscle force production after an intraperitoneal injection with TNF-α. However, the TNFα-induced force depression was counteracted by pre-treatment with the NOS inhibitor L-NAME [95]. Furthermore, TNF-α is also thought to regulate the production of iNOS, which have been persistently found in serum and synovial fluid of RA patients [96,97]. However, we and others have not been able to detect any changes in iNOS (or eNOS) levels in skeletal muscle afflicted by arthritis [12,23,24,98].

## Counteracting oxidative stress-induced muscle weakness in RA

RA is currently treated through a ‘treat-to-target’ approach, which means that the aim of the treatment of RA is disease remission [2]. The definition of disease remission has been determined by the European League Against Rheumatism (EULAR) and the American College of Rheumatology (ACR) [2,99,100]. To estimate if disease remission is accomplished, the severity of RA is measured through scoring systems that assess several factors, such as number of swollen and tender joints, erythrocyte sedimentation rate, and questionnaires that estimate the patient’s sensation of pain, fatigue and overall health [100]. A commonly used scoring system is the disease activity score (DAS) that classifies the state of RA into remission, low disease activity, moderate disease activity and high disease activity [2]. Disease-modifying anti-rheumatic drugs (DMARDs) are currently the first line treatment for RA patients and can have a broad immunosuppressive activity (conventional DMARDs, such as methotrexate and leflunomide) or specific by targeting pro-inflammatory cytokines, such as TNF-α or IL-6 (biological DMARDs, e.g. adalimumab and sarilumab) [2,99]. Although DMARDs are efficient in retaining the patient in a low state of disease activity, a study by Lemmey et al. explicitly showed that tight disease control of RA patients with DMARDs still fails to improve body composition and physical function [9]. Moreover, the patients included in our recent study were prescribed medication with a broad immunosuppressive activity in combination with anti-inflammatory treatments, but these patients were still significantly weaker [11]. This shows that muscle dysfunction cannot be counteracted by anti-inflammatory medication alone. Instead, pre-clinical studies in rodents have shown that specifically targeting oxidative stress in skeletal muscle can restore the force generating capacity in muscles afflicted with arthritis [23]. Thus, antioxidant treatment could potentially be a beneficial combinatory treatment to DMARDs to improve muscle function in afflicted patients.

### Targeting oxidative stress: endogenous and synthetic antioxidants

Oxidative stress evidently plays an essential role in the progression and development of RA, as well as in the loss of skeletal muscle strength. The endogenous antioxidant defense system is responsible for normalizing the ROS levels and thus protects us against oxidative stress. Therefore, it is not surprising that there are reports of RA patients exhibiting lower levels of endogenous antioxidants than healthy individuals [101–104].

Dietary and synthetical antioxidants have been tested in clinical trials for different diseases, but the outcome has often been inconclusive. One plausible reason to the failure of clinical trials is that general antioxidants have been used, which will allocate to the intracellular space and interact with surrounding molecules in a nonspecific and uncontrolled manner, including vitamin E, vitamin C and a recent attempt to reduce RA disease activity with N-acetylcysteine (NAC) [105–107]. Instead, in the attempt to counteract oxidative stress, one should aim to find a targeted antioxidant treatment that acts specifically at the sites where proteins identified as being negatively affected by the oxidative stress are present. In this regard, an superoxide dismutase (SOD) 2/catalase mimetic (EUK-134) that supposedly targets the mitochondria has shown promising effects in the attempt to prevent muscle weakness in rats with arthritis [23] and in a rodent model of pulmonary hypertension [31].

### Enhancing the endogenous ROS defense system by exercise

In addition to targeting the oxidative stress with specific synthetic antioxidants as an attempt to counteract muscle weakness, the endogenous antioxidants (e.g. SOD, catalase) can be stimulated by exercise, primarily endurance exercise [108,109].

In contrast with the chronic and exacerbated ROS production associated with the pathology of RA that results in cellular damage and skeletal muscle dysfunction [11,24], a transient low-to-moderate production of ROS during exercise is essential to promote cellular and muscular adaptations, including increased antioxidant levels (e.g., SOD, catalase, GPx), as well as up-regulation of key enzymes for β-oxidation and mitochondrial function [110,111].

Activation of redox-sensitive pathways, such as peroxisome proliferator-activated receptor gamma co-activator 1-α (PGC-1α)–nuclear respirator factor (NRF) 1/2 axis plays a pivotal role in exercise-mediated antioxidant and muscular adaptations [110,112]. For instance, exercised-induced ROS, mechanical stress and energetic demands lead to bursts of increased intramuscular levels of PGC-1α mRNA and protein, which after some delay results in activation and expression of genes related to mitochondrial biogenesis [113–115]. This includes NRF1 and mitochondrial transcription factor A (TFAM) that contribute to increased mitochondrial content and oxidative phosphorylation [113–115]. PGC-1α also promotes the activation and translocation of the nuclear factor erythroid 2-related factor 2 (Nrf2), an important regulator of several endogenous antioxidants, including thioredoxin reductase 1 (Txnrd1), glutamate-cysteine ligase (GCL) and heme oxygenase-1 (HMOX1) [112,116]. The expression of PGC-1α is negatively correlated with pro-inflammatory cytokine levels in chronic inflammatory diseases [117]. However, to our knowledge, the role of PGC-1α in the skeletal muscle dysfunction in RA has not been addressed yet, although its close homolog PGC-1β has been shown to be elevated in synovium of RA patients where it plays an important role in pro-inflammatory response through the activation of NF-kB transcription [118].

In addition to inflammatory disease, increased levels of intramuscular and circulating cytokines, such as IL-6 and TNF-α, have been observed after exercise [119]. In fact, inflammation is an acknowledged process in muscular repair and regeneration after exhausting or unaccustomed bouts of exercise [119–121]. For instance, transient post-exercise increase of IL-6 is associated with hypertrophic muscle growth and myogenesis, and beneficial effects on energetic metabolism [122,123]. Whereas, chronic exposure of IL-6 results in atrophy and muscle wasting [123,124]. The underlying molecular mechanisms of the pleiotropic and antagonistic effects of IL-6 remain to be clarified, but the current understanding of the field are thoroughly reviewed by Tuna and colleagues [125]. Moreover, TNF-α is involved in the pathology of RA where it is associated with muscle weakness [12,23] and in the response to exercise, for example, acute, strenuous exercise is associated with increased circulating levels of TNF-α, contributing to the inflammatory state that contributes to muscle adaptation after exhausting or unaccustomed bouts of exercise [119–121].

Inflammatory-induced pain may be a contributing factor to a sedentary lifestyle in RA patients, which could contribute to reduced muscle performance. However, it is possible that the resistance to perform physical activity for RA patients is also associated with a lack of education and information about the benefits of physical activity rather than an increased perception of pain by the RA patient. Indeed, besides reducing the perception of pain and fatigue, physical exercise interventions have been shown to decrease oxidative stress markers in plasma from RA patients [101,126,127]. Moreover, Mateen and colleagues have shown that hydrotherapy exercise combined with conventional DMARDs increased the activity of endogenous antioxidant enzymes (SOD and glutathione peroxidase, GPx), which coincided with lower MDA and carbonylation levels on proteins from the exercise group then the control group [101].

In future studies, it would be intriguing to further elucidate whether different type of exercises and exercise-mimetics can boost the endogenous ROS/RNS defense system and thereby counteract muscle weakness and thus improve physical function in patients with RA.

## Final remarks

Here we have reviewed intramuscular aspects that contribute to muscle weakness associated with RA and discussed possible underlying mechanisms and molecular players that likely play a role. Cytokines form the first line of defense of the innate immune system and often they induce complex signaling cascades that also influences redox signaling inside muscle cells. Specifically, IFNγ, TNF-α, OSM, IL-1β and IL-6 are observed to be elevated in RA and are known inducers of ROS/RNS. In addition, mitochondria, NOX, PLA and NOS are ROS/RNS sources and hence possible sites from where oxidative stress originate. These inducers and sources of ROS/RNS contribute to arthritis-induced muscle weakness; however, many of the molecular details of how this occurs remain to be elucidated, including the temporal and spatial signaling of ROS. Moreover, there is currently a serious deficit in clinical therapeutic approaches to counteract muscle weakness. However, the results presented here provide new leads for the development of such targeted treatments. For instance, identifying which ROS/RNS source that is primarily responsible for the excess production in skeletal muscle afflicted by arthritis, could be a big step forward toward defining a druggable target for a specific antioxidant treatment to counteract muscle weakness, enhance physical function and thus ultimately improve the quality of life of afflicted patients suffering from muscle weakness and fatigue.

## Open Access

Open access for this article was enabled by the participation of Karolinska Institute in an all-inclusive *Read & Publish* pilot with Portland Press and the Biochemical Society.

## Abbreviations

ACPA: anti-citrullinated protein antibody
IL: interleukin
MCP1: monocyte chemoattractant protein 1
NOS: nitric oxide synthases
NOX: NADPH oxidase
OSM: oncostatin M
PLA: phospholipase
RA: rheumatoid arthritis
RF: rheumatoid factor
ROS: reactive oxygen species
RNS: reactive nitrogen species
TNF-α: tumor necrosis factor-α
